# Complete plastome sequence from a cultivar of *Diospyros nigra* (J.F.Gmel.) M.R. Almeida (Ebenaceae): a nutritious fruit tree with high economic value cultivated in Hainan province, China

**DOI:** 10.1080/23802359.2022.2072243

**Published:** 2022-05-09

**Authors:** Xu Xin-Xin, Hong-Xin Wang, Da-Juan Chen, Xiu-Rong Ke, Zhi-Xin Zhu, Hua-Feng Wang

**Affiliations:** a Hainan Key Laboratory for Sustainable Utilization of Tropical Bioresources, College of Tropical Crops, Hainan University, Haikou, China; b Zhai Mingguo Academician Work Station, Sanya University, Sanya, China; c Sanya Nanfan Research Institute of Hainan University, Hainan Yazhou Bay Seed Laboratory, Sanya, China

**Keywords:** *Diospyros nigra*, Ebenaceae, genome structure, plastome

## Abstract

*Diospyros nigra* (J.F.Gmel.) M.R.Almeida is a rare tree in the family Ebenaceae. The species is native to South America, while having been introduced to Florida and Texas (USA), India, Java and Madagascar. Additionally, this species is distributed in Guangdong Province and the southwest portion of Hainan Province, China. Here, we report and characterize the complete plastome of a cultivar of *D. nigra.* The length of the complete plastome is 157,168 bp, including 131 genes consisting of 84 protein-coding genes, 37 tRNA genes and 8 rRNA genes. The plastome has the typical structure and gene content of angiosperms, including two inverted repeat (IR) regions of 26,095 bp, a large single copy (LSC) region of 86,610 bp and a small single-copy (SSC) region of 18,386 bp. The total G/C content of the plastome in *D. nigra* is 37.4%. The complete plastome sequence of *D. nigra* will make contributions to the conservation genetics of the species, as well as to phylogenetic studies in Ebenaceae.

## Introduction

*Diospyros nigra,* (J.F.Gmel.) Perrier 1996 is a rare tree in the family Ebenaceae. The species is native to Central and South America, including Belize, Colombia, Costa Rica, El Salvador, Guatemala, Honduras, Mexico (including the central, gulf, northeast, southeast, and southwest regions), Nicaragua, and Panamá. Recently *D. nigra* has been introduced into Florida (USA), India, Java, Madagascar, Texas (USA), as well as Guangdong and Hainan provinces of China. The fruit of *D. nigra* is edible, and a variety of biochemical reagents can be extracted from the trunk. Therefore, we report the complete plastome of *D. nigra* in this study, which will improve the quality of relevant functional, medical application and phylogenetic studies of Ebenaceae.

In this study, fresh leaves of *D. nigra* were sampled from the city of Qionghai, Hainan, China (110.46°E, 19.35°N). The leaves were placed in silica gel after collection. Total genomic DNA was extracted from dried leaf tissue using the cetyltrimethyl ammonium bromide (CTAB) protocol of Doyle and Doyle (1987).

A voucher specimen (voucher code: D.-J. Chen, X.-R. Ke, A64, HUTB, Wang et al., A64) and corresponding DNA were deposited in the Herbarium of the Institute of Herbarium of China National GenBank (code of herbarium: HUTB).

The experiment was carried out as reported in Zhu et al. (2018). Clean sequence data was assembled with GetOrganelle v1.7.5.0 (Jin et al. 2020). The plastome was annotated against the plastome of *D. mespiliformis* (MZ274088) using Geneious Prime v2021.1.1 (Biomatters Ltd, Auckland, New Zealand) and the annotation was corrected with DOGMA (Wyman et al., 2004).

Our results show that the plastome in *D. nigra* has a typical quadripartite structure of angiosperms with 157,168 bp, consisting of 26,095 base pairs for the two Inverted Repeats (IR) regions, 86,610 base pairs for the Large Single Copy (LSC) region and 18,386 base pairs for the Small Single Copy (SSC) region. The plastome consists of 131 genes, represented by 84 protein-coding genes (five of which are duplicated in the IR), 37 tRNA genes (seven of which are duplicated in the IR) and 8 rRNA genes (5 s rRNA, 4.5 s rRNA, 23 s rRNA, and 16 s rRNA) (four of which are duplicated in the IR). The total G/C content of the plastome of *D. nigra* was 37.4%, and the G/C content of the LSC, SSC, and IR regions were 35.4, 30.7, and 43.1%, respectively.

**Figure 1.**
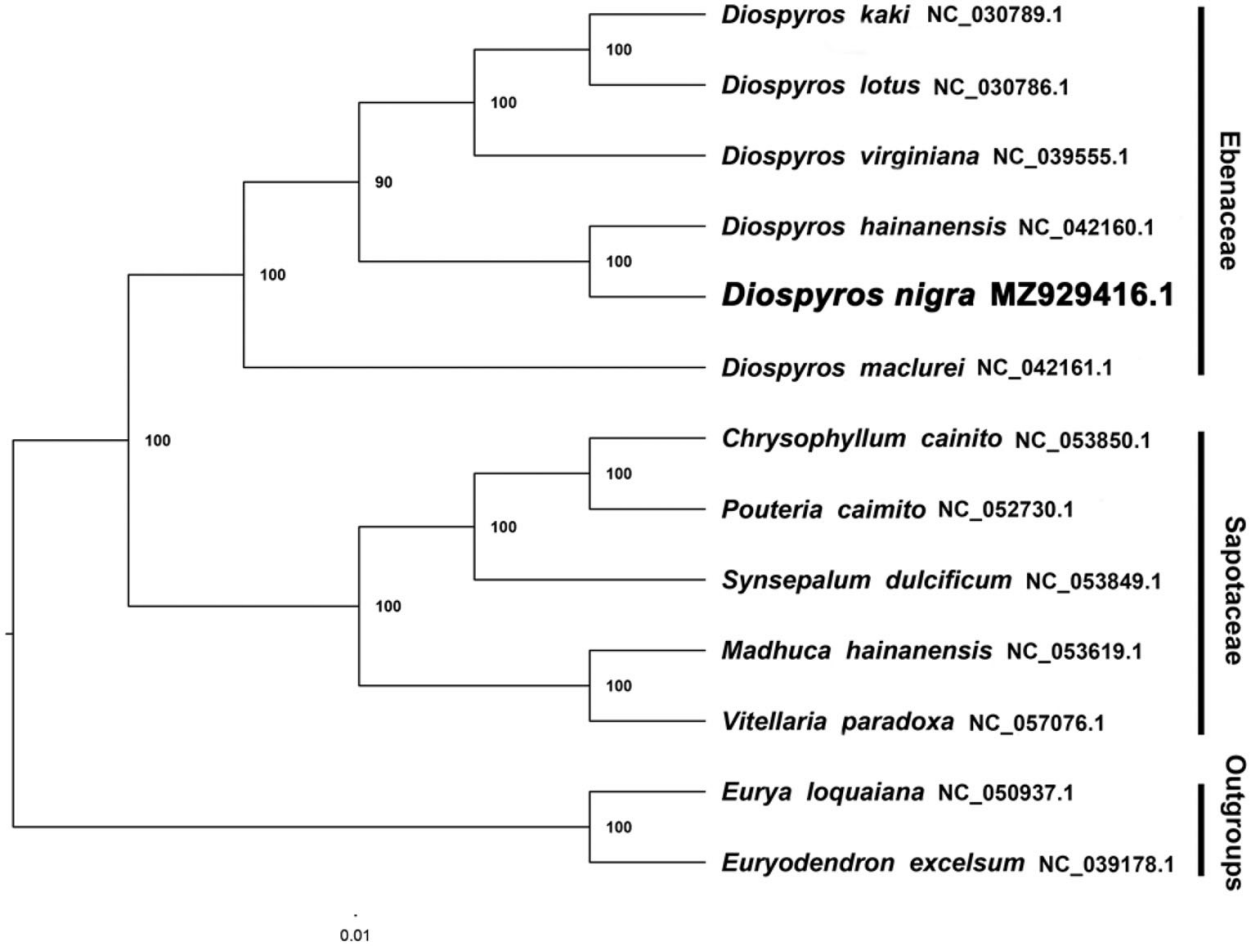
The maximum likelihood phylogeny recovered from 11 complete plastome sequences using RAxML.

We inferred a phylogenetic tree using Maximum Likelihood (ML) with CIPRES (http://www.phylo.org/portal2/). By reconstructing phylogenetic relationships based on the existing data of related taxa, the results show that *D. nigra* is more closely related to *Diospyros hainanensis* than other species included in this study (Figure 1). The current data show that most nodes in the plastome ML tree were highly supported. The presented plastome of *D. nigra* provides a great resource for promoting relevant conservation and phylogenetic investigations of Ebenaceae.

## Ethical approval

The study was approved by the institutional review board of Hainan University, Haikou, China. The collection of plant materials is carried out in accordance with guidelines provided by the Hainan University and Hainan province regulations. Field studies comply with Hainan province field work policy drafted by Hanan University (in Chinese), and the manuscript include a statement of appropriate permissions granted and licenses from related agencies of Hainan province. Voucher specimens are deposited in a public herbarium (HUTB) providing access to deposited material. Information on the voucher specimen and who identified it is included in the manuscript.

## Data Availability

The genome sequence data supporting the results of this study are available in the public database of GenBank of NCBI (https://www.ncbi.nlm.nih.gov/) with registration number MZ929416.1. The associated BioProject, SRA, and Bio-Sample numbers are PRJNA748537, SRR15533080 and SAMN20703177, respectively. A specimen was deposited at Hainan University (https://ha.hainanu.edu.cn/home2020/, H.-F. Wang and hfwang@hainanu.edu.cn) under the voucher number D.-J. Chen, X.-R. Ke, A64.

## References

[CIT0001] Doyle JJ, Doyle JL. 1987. A rapid DNA isolation procedure for small quantities of fresh leaf tissue. Phytochem. Bull. 19:789–15.

[CIT0005] Wyman SK, Jansen RK, Boore JL. 2004. Automatic annotation of organellar genomes with DOGMA. Bioinformatics. 20(17):3252–3255. 15180927 10.1093/bioinformatics/bth352

[CIT0006] Zhu ZX, Mu WX, Wang JH, Zhang JR, Zhao KK, Friedman CR, Wang HF. 2018. Complete plastome sequence of *Dracaena cambodiana* (Asparagaceae): a species considered “vulnerable” in Southeast Asia. Mitochondrial DNA B Resour. 3(2):620–621. 33474263 10.1080/23802359.2018.1473740 PMC7800030

